# Genome Sequence of Mushroom Soft-Rot Pathogen *Janthinobacterium agaricidamnosum*

**DOI:** 10.1128/genomeA.00277-15

**Published:** 2015-04-16

**Authors:** Katharina Graupner, Gerald Lackner, Christian Hertweck

**Affiliations:** a Leibniz Institute for Natural Product Research and Infection Biology (HKI), Department of Biomolecular Chemistry, Jena, Germany; b Friedrich Schiller University, Jena, Germany

## Abstract

*Janthinobacterium agaricidamnosum* causes soft-rot disease of the cultured button mushroom *Agaricus bisporus* and is thus responsible for agricultural losses. Here, we present the genome sequence of *J. agaricidamnosum* DSM 9628. The 5.9-Mb genome harbors several secondary metabolite biosynthesis gene clusters, which renders this neglected bacterium a promising source for genome mining approaches.

## GENOME ANNOUNCEMENT

The soft-rot disease of the cultured button mushroom *Agaricus bisporus* results from an infection with the Gram-negative bacterium *Janthinobacterium agaricidamnosum* (1). Despite its devastating disease outcome that accounts for substantial losses in agriculture, the pathobiology of the soft-rot disease has not been investigated in the past. Recently, we discovered that the cyclic lipopeptide jagaricin is involved in the soft-rot infection process (2). Moreover, jagaricin exhibits strong antifungal activity against major human pathogenic fungi (2).

The genome of *J. agaricidamnosum* DSM 9628 was sequenced using the 454 GS FLX Titanium system (282,254 reads) with an 8-kb paired-end library (405,849 reads) to a 24-fold coverage. The Newbler assembler (454 Life Science) was used for assembly of the sequencing reads. 167 contigs (*N*_50_ contig size 113,797 bp) were assembled into 9 scaffolds (*N*_50_ scaffold size 595,787 bp). Gene annotation was carried out by the IGS (Institute for Genome Science, University of Maryland, School of Medicine) prokaryotic annotation platform (3). The genome of *J. agaricidamnosum* has a total size of 5,949,001 bp, has an overall G+C content of 61%, and consists of 5,573 open reading frames, of which 4,327 (77.6%) were assigned a biological function.

In addition to the characterized jagaricin biosynthesis gene cluster (2), whole-genome sequencing of *J. agaricidamnosum* revealed a gene locus for violacein production (2, 4, 5) as well as several orphan natural product biosynthesis gene clusters: Three gene clusters coding for nonribosomal peptide synthetases (NRPSs), one hybrid NRPS-polyketide synthase (PKS) gene cluster, one putative siderophore biosynthesis gene cluster, and one bacteriocin biosynthesis gene cluster. This genome analysis highlights that such neglected bacteria can be a hidden source for novel secondary metabolites (6).

To date, seven genomes of *Janthinobacterium* spp. are accessible by the DDBJ/EMBL/GenBank databases, and five of them have been published (7–11). However, *J. agaricidamnosum* is the first pathogenic *Janthinobacterium* that has been sequenced. The other *Janthinobacterium* spp. sequenced so far have been isolated from water, glaciers, soil, and rhizosphere.

Insight into the genome of *J. agaricidamnosum* not only reveals a high potential to produce secondary metabolites, but it could also aid in investigating the mechanism of soft-rot infection.

### Nucleotide sequence accession number. 

The genome sequence of *J. agaricidamnosum* has been deposited in DDBJ/EMBL/GenBank under the accession no. HG322949. The version described in this paper is the first version.
